# Taking turns: bridging the gap between human and animal communication

**DOI:** 10.1098/rspb.2018.0598

**Published:** 2018-06-06

**Authors:** Simone Pika, Ray Wilkinson, Kobin H. Kendrick, Sonja C. Vernes

**Affiliations:** 1 Department of Primatology, Max Planck Institute for Evolutionary Anthropology, Deutscher Platz 6, 04103 Leipzig, Germany; 2 Department of Comparative Biocognition, Institute of Cognitive Science, University of Osnabrück, Osnabrück, Germany; 3 Department of Human Communication Sciences, University of Sheffield, Sheffield, UK; 4 Department of Language and Linguistic Science, University of York, York, UK; 5 Neurogenetics of Vocal Communication, Max Planck Institute for Psycholinguistics, Nijmegen, The Netherlands; 6 Donders Institute for Brain, Cognition and Behaviour, Radboud University, Nijmegen, The Netherlands

**Keywords:** human language, language evolution, animal communication, turn-taking, duets, antiphony

## Abstract

Language, humans’ most distinctive trait, still remains a ‘mystery’ for evolutionary theory. It is underpinned by a universal infrastructure—cooperative turn-taking—which has been suggested as an ancient mechanism bridging the existing gap between the articulate human species and their inarticulate primate cousins. However, we know remarkably little about turn-taking systems of non-human animals, and methodological confounds have often prevented meaningful cross-species comparisons. Thus, the extent to which cooperative turn-taking is uniquely human or represents a homologous and/or analogous trait is currently unknown. The present paper draws attention to this promising research avenue by providing an overview of the state of the art of turn-taking in four animal taxa—birds, mammals, insects and anurans. It concludes with a new comparative framework to spur more research into this research domain and to test which elements of the human turn-taking system are shared across species and taxa.

## 1. Introduction

Language—the most distinctive human trait—remains a ‘mystery’ [1] or even a ‘problem’ for evolutionary theory [2,3]. Spoken languages can be characterized by two unique characteristics—a rich learned acoustic portfolio, and the predisposition to combine basic linguistic units into complex acoustic structures [4]. Languages differ at every level of construction, from the sounds, to syntax, to meaning embodying an unrivalled complexity, flexibility and expressivity combined with an unparalleled inter-group variation [5]. Traditionally, comparative studies aiming to unravel the evolutionary trajectory of language have tried to pinpoint the key modalities involved (gestures, vocalizations, combinations of gestures and vocalizations; [6,7]), and/or the underlying complexity in relation to production, usage and comprehension [8]. Recent advances in the fields of Cognitive Sciences, Genetics, Linguistics and Neurosciences, however, suggest that language is a relatively new invention composed of layers of abilities of different types and different antiquity [5,7]. Unpeeling these layers should enable us to understand which distinctive mechanisms were already in place when language first evolved from the communication systems of non-human primates.

In light of this view, an increasing amount of research attention has lately been devoted to the turn-taking system for conversation [9]. It is characterized by a reciprocal exchange of alternating, short and flexible turns between two or more interactants, is used universally across languages and cultures [10], and is based on specific properties [9]. Turn-taking skills develop earlier in ontogeny than gestural and linguistic competence [11], and show some signs of phylogenetic parallels in all clades of the primate lineage [5]. Levinson & Holler ([5], see also [12]) thus proposed that turn-taking may bridge the apparent gulf between the articulate human species and our non-articulate primate cousins. This hypothesis challenges the predominant view in the field, seeing language as part of a larger uniquely human adaptation for cooperation and cultural life in general [13]. The empirical pillars on which both hypotheses rest are, however, surprisingly weak: we know remarkably little about turn-taking systems of non-human primates and other animals, and methodological confounds have often prevented meaningful cross-species comparisons. Thus, the extent to which cooperative turn-taking is uniquely human or represents a homologous (by shared inheritance) and/or analogous (by parallel evolution) trait is currently unknown. A bias towards purely experimental set-ups and specific communicative modalities, such as the vocal modality, also hampers our understanding of turn-taking systems across different animal species [14,15]. These issues severely impair our understanding of this critical phenomenon, its phylogenetic history and the cognitive underpinnings enabling language to proliferate.

The present paper will draw attention to this promising research avenue by providing an overview of the state of the art of turn-taking in non-human animal communication. As there has been a tremendous number of publications on temporally coordinated signalling—and we can only cite some of them—we have restricted our selection of citations to publications that either cite secondary literature or are exemplary for general phenomena. We will briefly define the predominant terms used, explain the main functional hypotheses proposed, and then discuss their implications for current findings in four animal taxa—birds, mammals, insects and anurans. We conclude that a systematic quantitative comparison of a representative range of turn-taking skills among a single set of human and non-human animal individuals is needed to test the hypotheses of Levinson & Holler [5,12] and Tomasello [13]. To instigate such a comparison, we present a new framework enabling systematic, quantitative assessments of turn-taking abilities across species and taxa. We hope that this framework will spur more quantitative comparative work (and potentially falsify our claims), and shed light on the question of whether turn-taking has been the ‘small change’ that made a big difference in human history.

## 2. Turn-taking and related phenomena

The turn-taking system for conversation [9] applies equally to dyadic and multi-person interactions and is structured and organized according to set principles: alternating, and often relatively short, turns of varying size and order are exchanged between speakers, with only one party normally talking at a time. Speakers construct their turns out of units whose structure allows the next speaker to anticipate their completion. Turn transfer occurs at such points of completion, and turn-allocation works via specific techniques to minimize the temporal gaps between turns (≈200 ms [10]). This communicative exchange is seen as a fundamentally cooperative enterprise [16], involving elements such as ‘who should talk or move or act next and when should they do so’ ([17], p. 71). It allows interactants to coordinate turn allocation [17], avoid overlap (e.g. [18,19]) and inform others of things helpfully and/or share gossip freely [13], among a myriad of other social actions [20].

In non-human animal studies, multiple and not always mutually exclusive terms have been used to describe coordinated—and not solely communicative—exchanges between interactants involving alternating turns (box 1). Different terminology has been applied to refer to the same phenomenon (e.g. duetting and antiphonal calling) and the same terminology to depict different phenomena (e.g. duetting), while some terms are not mutually exclusive (e.g. turn-taking versus antiphonal calling or duetting). As this paper is concerned with phenomena most closely related to temporally coordinated turn-taking in human conversation, we do not review findings on chorusing, which is lacking in fixed time latencies [31].

Box 1. **Historical definitions of coordinated communicative exchanges**

  *Duetting*. This term—sometimes also referred to as dialogue [21,22] or calling songs in insects [23]—traditionally concerns acoustic interactions between two partners of the opposite sex [24]. While some scholars use the term to denote only loosely coordinated behaviours [25], others emphasize the predictable and stereotyped temporal association between an initiating call and its reply [24,26]. Some authors restrict the use of the term to the exact synchronization of identical notes [26], or overlapping bouts of vocalizations [27–29], while others also embrace both airborne and substrate acoustic signals (e.g. antiphonal tapping in woodpeckers), non-acoustic movements (*pas de deux*; [30]) and bioluminescence [24].

*Chorusing*. This term refers either to a cacophony of sounds or to the synchronous production of the same call type by more than two individuals [31].

*Antiphonal singing/song*. This term denotes a specialized form of duetting, in which one member of the pair starts a song that is then continued by the other member. The second member may complete the song or the members of the pair may take turns until completion [32].

*Antiphonal calling*. This term—sometimes also referred to as call-and-response—is defined as a minimum number of two individuals of any sex and/or age combination producing a vocalization in response to a preceding call [31]. Some authors use the term exclusively to denote vocalization exchanges involving the same call type only [33].

*Turn-taking*. This term was traditionally restricted to human spoken conversation but has recently been extended to other modalities and species. It denotes the orderly exchange of purely communicative signals or behaviours (e.g. peek-a-boo games in humans) between individuals characterized by principles for the coordination of turn transfer, which result in observable temporal regularities. The communicative signals delivered by turns can vary, as can the size and the order of turns, and techniques used to allocate turns to specific individuals [34]. Some scholars see turn-taking as an extension of ‘duetting-like’ vocal coordination to any conspecific [35].

## 3. The function of turn-taking^1^

Although temporal coordination in animal communication has attracted interest over several decades, no clear picture has yet emerged as to why individuals exchange signals. The explanations put forward generally have not distinguished between the function of signal exchange as such and the function of the exchange of specific signals (e.g. contact calls), though the motivations for these may differ. The earliest hypotheses on the function of duets^2^—inspired by the extraordinary precision of antiphonal singing in tropical bird species—focused on mutual recognition, maintenance of contact between partners, as well as mutual stimulation, reassurance after disturbance and territory defence [36,37]. In the 1980s, these hypotheses were challenged by Wickler ([25], following Armstrong [38]) based on the observed linkage between duetting and monogamy in birds and primates. Wickler tried to explain why temporally coordinated bird songs should be more effective than a solo song. In his view, bird duets function to strengthen the pair-bond by (i) maintaining contact between partners, (ii) synchronizing reproductive physiology, or (iii) advertising mated status. The resulting ‘coyness’ hypothesis postulated that pair-specific duets are costly strategies, because a high degree of song coordination between pairs is likely to take time and investment. New partners thus need to invest a lot of time learning to duet with the partner, deterring philanderers and making desertion less common [25,30]. In the 1990s, Levin [39] argued that duetting might be a consequence of conflict between the sexes. Recent reviews [27,40] suggest that duets can be multifunctional, including joint-resource defence, signalling commitment, maintaining contact, ensuring reproductive synchrony and mate-guarding. In addition, functions may differ between the sexes, can involve elements of both cooperation and conflict, and/or serve different functions in different circumstances [40,41].

In stark contrast, relatively little research attention has focused on the function of duets in amphibians and insects, and antiphonal exchanges in monogamous and polygamous living societies. The few existing studies on amphibians (*Anura*) and different orders of insects (*Orthoptera, Plecoptera, Hemiptera, Neuroptera)* suggest that the primary function of duets is to enable copulation by acting as mate-location devices [24,42]. Antiphonal exchanges may have partially similar functions as duetting [30], but may also be used to signal social rank and individuality, for coordination, individual recognition, maintenance of social bonds, social cohesion, social integration, and territory defence [25,29,43–48]. Furthermore, virtually nothing is known about the function of turn-taking in human societies [9,12].

## 4. Turn-taking in birds

Communicative vocal interactions of birds have been intensively studied for more than 50 years. To date, more than 360 species producing vocal duets have been reported [32,40,49]. In this section, we will focus on key aspects and common themes commonly investigated in communicative exchanges of birds including the type of signal used, the time window/temporal relationships (for definition, see Section 9 element (C); [9]), and the avoidance of overlap.

Overall, birds use a large variety of different signals across species, which range from simple calls (e.g. ka-ka; large-billed crow, *Corvus macrorhynchos*) [50] to extended songs (e.g. lesser skylark, *Alauda gulgula*) [51]. Interactions may involve each bird producing the same or a different call/song in response to the initiating vocalization. These interactions take place in different ways, with some species singing the same song in unison [30], while others coordinate their vocal output to produce different components of the same song [52], or engage in countersinging (where a second bird sings a coordinated but overlapping song) [18]. Some bird species, such as nightingales (*Luscinia megarhynchos*), perform ‘song matching’ (where the bird responds with the same song) or ‘vocal supplementing’ (where the bird responds with a different, but an appropriate continuation of the initial song) [18].

Research on the time window of avian vocal interactions has focused predominantly on duetting, while investigations into the time window of call exchanges in non-duetting species are relatively rare [50]. Information on the temporal precision of duets is available for 33 species across five orders (galliformes, gruiformes, psittaciformes, piciformes and passeriformes), with most research attention devoted to the order Passeriformes (which includes oscine passerines (songbirds) [40]). Temporal precision in most of these species is relatively high, with latencies between notes ranging from less than 50 ms [53] to 200 ms [40].

Analyses of time-specific relationships within vocal exchanges provided evidence that birds listen and respond to each other and show substantial flexibility in their temporal adjustments [18]. For instance, territorial common nightingales are able to precisely tune their song onset latencies with a peak of approximately 1 s after a neighbour has terminated his song [54]. Results from play-back experiments show that individuals are able to flexibly adjust and shift their latency peaks to account for changes in song duration of stimulus songs and to avoid overlap [54]. The phenomenon of overlap avoidance has been widely documented in several bird species, with the most detailed investigations focusing on nightingales, lesser skylarks [55] and large-billed crows [50]. Avoidance of overlap with regards to development has been studied in barn owls (*Tyto alba*) [56] and European starlings (*Sturnus vulgaris*) [57]. Nest-mates of European starlings, for example, exchange calls already very early in ontogeny in the absence of their parents with simultaneous calls occurring below chance level [57]. This finding suggests that distinct time windows may either be learned early, or represent (partially) an innate mechanism (see also the section on non-human primates below).

In addition to avoidance of song overlap, birds have also been observed to adopt two additional roles: they either follow their temporal self-program—called ‘autonomous songsters'—or start their songs sometime before (preferentially 1 s after song onset) a neighbour has finished singing—called ‘overlappers’ [18,54]. This diversity of behaviour represents flexible interaction strategies in some species and species-specific preferences in others. For instance, nightingales adopt different interaction roles in relation to season and social context [18]. By contrast, black-capped chickadees (*Poecile atricapillus*) favour overlap, and European starlings prefer overlap avoidance [58–60]. If overlap occurs, individuals become silent or fly away [60], suggesting that overlapping may be treated, in this species, as a violation of socially accepted rules of turn-taking [60]. It has also been speculated that the overlap itself carries communicative information such as signalling aggression or displaying dominance status [61] or results in direct fitness benefits [62]. For example, a study on quails (e.g. *Lophortyx californicus*) showed that males masking their females' identity prevented other unmated males from mating [62].

In addition, temporal patterns of vocal interactions seem to be tightly linked to a species’ social structure [60]. For example, a study on closely related species of Sturnids (African pied starling, *Spreo bicolor*; Cape glossy starling, *Lamprotornis nitens:* red-winged starling, *Onychognathus morio;* pale-winged starling, *Onychognathus nabouroup*) showed that the degree of territoriality highly influenced temporal relationships: the more communal the species, the more song overlap and choruses were observed during close-range interactions [60].

In sum, the use of distinct time windows in birds differs between but also within species ranging from overlap avoidance—representing a characteristic element of human turn-taking [9]—to the strategy of overlapping.

## 5. Turn-taking in mammals

Research into turn-taking propensities of mammals is strongly biased towards non-human primates. Within the order of primates, studies have nearly exclusively been focusing on vocal exchanges of pair-bonded, and/or family living species (e.g. [28,48,63]). Recently, researchers have also started to investigate turn-taking skills in the gestural modality [34,64], and expanded the research angle onto species living in multi-level [65] and fission–fusion societies [34].

### (a) Non-human primates

Some signs of turn-taking have been documented in all the major primate branches [12]: prosimians (e.g. *Lepilemur* spp. [66]; *Tarsius spectrum* [67]), New World monkeys (*Callicebus cupreus* [29]; *Callithrix jacchus* [63]; *Cebuella pygmaea* [48]; *Saimiri* spp*.* [65]), Old World monkeys (*Cercopithecus campbelli* [68]; *Theropithecus gelada* [69]), smaller apes (*Hylobates* spp*.* [28,70]) and great apes (*Gorilla gorilla; Pan paniscus; Pan troglodytes; Pongo abelii* [34,64,71]). The vast majority of research has focused on the structure and function of duets in monogamous primate species (e.g. indris *Indri indri*; gibbons *Hylobates* spp*.*; Mentawi langurs *Presbytis potenziani*; Titi monkeys *Callicebus cupreus* [28,29]), and the antiphonal call exchanges of a distinct clade of New World monkeys, the Callitrichids (e.g. [48,63]). These studies provided evidence that duets are initiated by both communication partners, are pure in tone (i.e. all of the sound energy is compressed into a narrow frequency band), and show manifold diversity [28]. In stark contrast, antiphonal call exchanges are relatively short and most often composed of single call types only (e.g. phee-calls of common marmosets [72]). Similar to antiphonal turn-taking in pair-bonded and/or family living species, turn-taking in polygynous societies seem to occur mainly between affiliated individuals [65]. Detailed studies on call exchanges of members of the Callitrichid family provide evidence for reciprocal coordination of vocal output [63], and sequential ‘conversational’ structuring [48]. In addition, studies investigating the temporal relationships underlying turn-taking exchanges showed considerable between-species variability ranging from approximately 500 ms in Saimiri monkeys (*Saimiri sciureus*) [73] to 3000–5000 ms in common marmosets (*Callithrix jacchus*) [63]. Although the development of turn-taking in non-human primates is relatively unexplored, studies on common marmosets imply that the use of antiphonal turn-taking is learned during ontogeny and actively guided by parents. For instance, parents responded differently to overlapping calls of their offspring compared to calls that did not overlap ([5] but see [74]). In addition, parents were more likely to interrupt inappropriate call types produced in response to a specific call (e.g. a twitter in response to a phee-call) than appropriate ones (e.g. phee-call in response to a phee-call) ([75] but see [74]).

Furthermore, spontaneous cooperative turn-taking has been observed in communicative gestural interactions of great apes in both captive and natural environments [34,76]. For instance, focusing on a specific sequential environment—joint travel initiations between mother-infant dyads—Fröhlich and colleagues showed that bonobos (*Pan paniscus*) and chimpanzees (*Pan troglodytes*) establish participation frameworks and adjacency pair-like sequences (for definition, see Section 9 element (D); [9]). Gestural responses can match the temporal relationships observed in human speech (bonobos: 200–1400 ms; chimpanzees: 200–1800 ms) but can also be significantly longer.

### (b) Non-primate mammals

Outside of the primate order, vocal turn-taking has been studied in four distantly related mammalian groups, cetaceans, bats, elephants and mole rats.

The most research attention has been devoted to cetaceans and provided evidence that vocal exchanges facilitating social interactions occur in a number of species including beluga whales (*Delphinapterus leucas*) [77], bottlenose dolphins (*Tursiops truncatus*) [78], killer wales (*Orcinus orca*) [79], southern right whales (*Eubalaena austrialis*) [80] and sperm whales (*Physeter macrocephalus*) [81]. Bottlenose dolphins represent the best studied cetacean species, partly facilitated by their relatively small size and relative frequency in captivity. Dolphins produce characteristic signature whistles that are used in coordinated vocal interactions and seem to facilitate individual recognition and maintenance of group cohesion. Isolated dolphins in captivity use alternating whistles with minimal overlap when two or more dolphins can interact (physically or only acoustically) [78,82–84] (although in the wild, overlap appears more frequent, see [85]). Time windows of vocal turns between interacting individuals were generally less than 1 s [47,83,84]. Observations on dolphins in their natural environments showed that exchanges of whistles commonly precede an animal joining a group [86]. Similarly, southern right whales exchange a specific call type—the ‘up‘ call—during approach and integration into a group. Pairs of dolphins in captivity have also been observed to partake in duets characterized by closely matched frequencies and timing of whistles. They are also able to swap between alternating (antiphony) and duetting within the same train of vocalizations [78]. Similar to the signature whistles of dolphins, beluga whales use burst pulse sounds. These calls are predominantly produced within a time window of approximately 1 s following a burst pulse sound produced by a conspecific [77]. They thus mirror the timing of signature whistle exchanges in dolphins. By contrast, and possibly due to living in stable rather than fission–fusion societies, beluga whales, killer whales and sperm whales exchange group-specific calls (characteristic for single groups; [79,81,87]). These calls are used in an antiphonal manner and are much more likely to occur within a time window of approximately 5 s [79]. Furthermore, call types are frequently matched (responding to the first call with the same call type) by conspecifics [79], suggesting that the vocal behaviour of group members highly impacts upon timing and call type choice. Similarly, sperm whales exchange sequences of broadband clicks (codas) with either temporal gaps of 2 s or by using overlapping codas, and also show call matching of the original coda type [81].

Much less information is available regarding turn-taking in bats, elephants and mole rats. Bats engage in antiphonal calling between adults (white-winged vampire bats; *Diaemus youngi*) [43] or between mothers and their offspring (young pups not capable of flight) in the families of Molossidae, Vespertilionidae, Phyllostomidae and Emballonuridae [44,88–92]. Temporal relationships have so far only been studied in the white-winged vampire bats, ranging from 300–350 ms [43]. Female elephants (*Loxodonta africana*) exchange vocalizations, such as low-frequency rumbles, to respond to calls from other females [93,94]. A response is most likely when the interacting females have strong social relationships [93,94], with call exchanges often resulting in closer proximity between participating animals [46]. Naked mole rats (*Heterocephalus glaber*) are one of the few eusocial mammal species, and use their most common vocalization type—the soft chirp—antiphonally with a latency of approximately 400 ms [45,95].

## 6. Turn-taking in insects

Research into communicative exchanges of insects has focused on five different orders: the Choleoptera, the Hemiptera, the Neuroptera, the Ortoptera and the Plecoptera [22–24,96]. The signal producing mechanisms are very diverse and range from vibration, percussion, stridulation, over click mechanisms, air expulsion, to bioluminescence [22,97]. The first signal of a given interaction is—in contrast to the more flexible duets of birds and mammals [98]—always initiated by males, with mechanisms often differing consistently across the sexes [24]. For instance, the sounds or substrate vibrations of males of many homopteran cicadellids and cicadas are produced by a tymbal, while the females respond by using vibrations created by movements of the wings [24]. The length of the initiating call is highly variable within-species, while the distinctive temporal pattern and the time window between signal and female reply are often species-specific [99]. Males of a species that initiate duets via long complex calls often insert a trigger pulse at the end of the call [100], which may act as a cue for the female to reply [24]. The variability of signal interaction between the sexes is manifold, ranging from brief exchanges (e.g. stonefly, *Eucoptura xanthenes*) to relatively complex sequences involving females alternating their replies between the pulsed phrases of the male signal (e.g. North American katydid, *Amblycorypha parvipenni*) [24]. Temporal relationships vary from extremely short intervals (e.g. 15 ms, blackwinged saw bush-cricket *Ancistrus nigrovittata*; 20–30 ms, speckled bush-cricket *Leptophyes punctatissima*) to even 850 ms in species relying on bioluminescent systems (e.g. Photinus firefly *Photinus greeni* [96]).

## 7. Turn-taking in anurans

In anurans, turn-taking mainly takes place in the form of antiphonal advertisement calls by males to attract females and has been observed in most groups of frogs. Males producing vocalizations in close distance to each other (or in response to playbacks) will typically become temporally entrained such that overlap is avoided. Calls thus occur within defined time points after the completion of another call [101–103]. For example, in green frogs (*Rana clamitans*) calls from different males are spaced at intervals of 2–10 s and rarely overlap [104]. Males of the Sri Lankan tree frog (*Philautus leucorhinus*) engage in vocal matching which appears to be based on the nature of the rival's call rather than simply being an example of vocal stereotypy [105]. A small number of species has been observed where overlap is typical [103]. For example, American toad males (*Bufo americanus*) engage in synchronous, or near-synchronous, overlapping of calls [103,104]. And, while unusual, males and females of some species also engage in duets. In American clawed frogs (*Xenopus laevis*), for example, females may produce a ‘rapping’ vocalization in response to male calling which is then responded to with a male answer call [106].

## 8. Conclusion

Overall, direct comparisons of turn-taking skills of non-human animals in relation to language origins (but also social communication and communication in general) are highly constrained by lack of data, the application of different terms, methodological designs (observational versus experimental paradigms) and study environments (captivity versus natural environments). Furthermore, investigations have so far mainly been focusing on single call types (e.g. phee-call in common marmosets) or songs (e.g. great-calls in gibbons) of species, limiting an in-depth understanding of the variability and underlying cognitive flexibility of turn-taking systems found in the animal kingdom.

To date, the parameters tested across different taxa and species to infer the organization of non-human animal turn-taking have mainly concerned a single key element of full-blown human turn-taking—the time window. Hence, the temporal adaptation and alteration of signal production seems to be a basic element of sociality and communication in general, and may have been the first step in the evolution of turn-taking systems. However, it is currently virtually impossible to evaluate whether time windows across species and taxa are indeed similar phenomena or differ because different definitions, methodologies and signal types (differing in form and function) have been investigated.

Most progress concerning an in-depth understanding of the degree of similarity between human and non-human communicative turn-taking systems has been made by studies taking into consideration Sacks *et al*.’s [9] systematics for the organization of human turn-taking. For instance, Rossano [76] and Fröhlich *et al*. [34] investigated gestural interactions of great apes with a special focus on turn-allocation techniques, distinct time windows and adjacency pair-like structures. They showed that bonobos and chimpanzees use gaze and distinct proximity patterns to allocate turns. In addition, both species have species-specific time windows, and are able to form adjacency pair-like structures (e.g. a carrying request resulting in being carried).

Future studies should push this approach even further by testing whether the most crucial hallmarks of human conversational turn-taking can be found in turn-taking systems of other animals: who should communicate, move or act and when should interactants do so [17]. Such an unprecedented rigorous test of turn-taking skills will enable us to gain insight into the layers shared across species and taxa and the cognitive complexity underlying specific elements of the turn-taking system. For instance, rhythmic signalling in many insect and anuran species is controlled by a central nervous system oscillator that may be inhibited, and reset by an acoustic stimulus such as a competitor's call ([103,104,107], but see [108]). There is no response *per se* to preceding signals, while in contrast species-specific time windows in monkey species are learnt [68], with individuals taking into consideration sex, age and rank of recipients [75,109–111].

## 9. The comparative turn-taking framework

The new framework enabling comparative, systematic, quantitative assessments of turn-taking abilities centres on four key elements characterizing human social action during conversation:

(A) Flexibility of turn-taking organization
(B) Who is taking the next turn?
(C) When do response turns occur?
(D) What should the next turn do?

The first element—flexibility of turn-taking organization (A)—refers to the phenomena of varying size and ordering of turns and intentionality involved in human turn-taking sequences [9]. The element mirrors the ability to voluntarily change and adjust signals/actions and thus the degree of underlying cognitive flexibility. It can be operationalized by quantifying the number, frequency and degree of repetition of signals and actions produced in turn-taking events, their combination (e.g. A-B-A; A-B-C), distribution of roles between participants (e.g. role reversal), and intentionality involved (e.g. goal persistence, sensitivity to the social context) [34,112,113].

The second element—who is taking the next turn (B)—concerns who can or should produce the next signal and includes techniques for allocating turns to individuals or parties [9]. Parameters should involve (i) body orientation towards recipient(s), (ii) gaze direction of signaller, (iii) response waiting, and (iv) whether recipient(s) can perceive the signal (e.g. being in the visual or auditory field).

The third element—when do response turns occur (C)—addresses the *time window* or *temporal relationship* between an initiating turn and the response turn [10,24]. Since the normative timing of signal exchanges may differ across species, modalities, and transmission medium, a first mandatory step should be to establish typical time windows for a given species (see [34] for ideas to operationlize this element).

The fourth element—what should the next turn do? (D)—concerns one of the most fundamental structures in the organization of human conversation: *adjacency pairs* [114]. An adjacency pair can be recursively reproduced [115] and expanded in conversation and—in its minimal, unexpanded form—is composed of two turns, by different participants, that are adjacently placed, and are relatively ordered into *first pair parts* (actions that initiate some exchange, e.g. requests), and *second pair parts* (responsive actions, e.g. grants) [114]. This element can be operationalized by testing whether subsequent turns qualify as adjacency pairs involving predictable signal-response sequences (e.g. a request gesture is typically responded with a granting signal; a call is typically responded with the same call type, e.g. common marmosets) [74,116].

## 10. Empirical desiderata

A major avenue of future research is to use the comparative turn-taking framework to characterize the turn-taking phenotype of a wide variety of primate species. This could be done through systematic testing of carefully chosen representatives of more than 50 genera of primates, which should then enable us to map out cladistically the evolution of primates' turn-taking skills and systems. Furthermore, recent findings on language competence and cognitive skills of members of the parrot and corvid family [117–119] have put into question the assumed simple inverse correlation between language-readiness and genetic distance from humans. Although avian and primate brains differ significantly in size, structure, and neuron numbers, similar principles of organization are evident, reflecting a case of convergent evolution in relation to mental processes [120,121]. Examples of convergent evolution in distant-related species can, therefore, provide important clues to the types of problems that particular morphological or behavioural mechanisms are ‘designed’ to solve. Furthermore, in order to claim that particular components of human language are unique to humans, data indicating that no other animal has this particular trait is required.

Such an unprecedented, systematic comparative approach will empower us to test whether cooperative turn-taking represents the most ancient infrastructure of the language system and has been the ‘small change’ that made a big difference in human history. This new field of comparative turn-taking will thus shed light on one of the ‘hardest’ problems in science [3] by testing whether turn-taking had profound downstream effects on human culture and cooperation, and laid the foundation for the evolution of language.

## Endnotes

1 From here on, the term ‘turn-taking’ refers to all temporal coordinated phenomena introduced.

2 From here on, the terms ‘duet’ and ‘antiphonal’ mirror the use of these terms in the reviewed studies, but do not reflect an assessment of the authors.

## Data accessibility

This article has no additional data.
